# A predictive model for healthcare coverage in Yemen

**DOI:** 10.1186/s13031-020-00300-1

**Published:** 2020-08-05

**Authors:** Mark P. Suprenant, Anuraag Gopaluni, Meredith K. Dyson, Najwa Al-Dheeb, Fouzia Shafique, Muhammad H. Zaman

**Affiliations:** 1 Department of Biomedical Engineering, Boston University, Boston, MA USA; 2 UNICEF, Yemen Country Office, Aden, Jordan; 3 UNICEF, Yemen Country Office, Sana’a, Yemen; 4 UNICEF, NYHQ, Aden, Yemen; 5 Howard Hughes Medical Institute, Boston University, Boston, MA USA

**Keywords:** Yemen, Markov model, Computational modeling, Diarrheal disease, Childhood illnesses, Healthcare coverage

## Abstract

**Introduction:**

The ongoing war in Yemen continues to pose challenges for healthcare coverage in the country especially with regards to critical gaps in information systems needed for planning and delivering health services. Restricted access to social services including safe drinking water and sanitation systems have likely led to an increase in the spread of diarrheal diseases which remains one of greatest sources of mortality in children under 5 years old. To overcome morbidity and mortality from diarrheal diseases among children in the context of severe information shortages, a predictive model is needed to determine the burden of diarrheal disease on Yemeni children and their ability to reach curative health services through an estimate of healthcare coverage. This will allow for national and local health authorities and humanitarian partners to make better informed decisions for planning and providing health care services.

**Methods:**

A probabilistic Markov model was developed based on an analysis of Yemen’s health facilities’ clinical register data provided by UNICEF. The model combines this health system data with environmental and conflict-related factors such as the destruction of infrastructure (roads and health facilities) to fill in gaps in population-level data on the burden of diarrheal diseases on children under five, and the coverage rate of the under-five sick population with treatment services at primary care facilities. The model also provides estimates of the incidence rate, and treatment outcomes including treatment efficacy and mortality rate.

**Results:**

By using alternatives to traditional healthcare data, the model was able to recreate the observed trends in treatment with no significant difference compared to provided validation data. Once validated, the model was used to predict the percent of sick children with diarrhea who were able to reach, and thus receive, treatment services (coverage rate) for 2019 which ranged between an average weekly minimum of 1.73% around the 28th week of the year to a weekly maximum coverage of just over 5% around the new year. These predictions can be translated into policy decisions such as when increased efforts are needed to reach children and what type of service delivery modalities may be the most effective.

**Conclusion:**

The model developed and presented in this manuscript shows a seasonal trend in the spread of diarrheal disease in children under five living in Yemen through a novel incorporation of weather, infrastructure and conflict parameters in the model. Our model also provides new information on the number of children seeking treatment and how this is influenced by the ongoing conflict. Despite the work of the national and local health authorities with the support of aid organizations, during the mid-year rains up to 98% of children with diarrhea are unable to receive treatment services. Thus, it is recommended that community outreach or other delivery modalities through which services are delivered in closer proximity to those in need should be scaled up prior to and during these periods. This would serve to increase number of children able to receive treatment by lessening the prohibitive travel burden, or access constraint, on families during these times.

## Introduction

Since early 2015, Yemen has been embroiled in a civil war resulting in a humanitarian crisis [1]. Prior to the start of the conflict, it was estimated that about half of Yemeni citizens had access to healthcare facilities [2]. Due to a combination of the direct effects of the conflict (active fighting, insecurity, population movement and damage to health facilities and other health infrastructure) and the secondary effects of economic crisis and collapsing public systems, just over 60% of the country’s medical facilities were fully operational by 2016 based on the 2016 Health Resources Availability Monitoring System in Yemen (HeRAMS) and estimations for how partially functional facilities compare to fully operational ones [3]. Given the destruction of water and sanitation infrastructure leading to poor water quality and environmental health conditions, the environment is highly conducive to an increase in diarrheal diseases [4]. Internally displaced persons (IDPs) often live in even worse conditions than the general population, elevating their risk further. The causes of diarrhea among children may be viral, bacterial or parasitic. In any of these cases, it is well accepted that zinc and oral rehydration salt solution (Zn/ORS) is the best and most cost effective course of treating the resulting dehydration and reducing the duration of a diarrhea episode, costing an average of $0.50 per dose [5, 6]. In line with WHO guidelines, Yemen’s national protocol for treating diarrheal disease among children under five calls for use of Zn/ORS for all cases and use of antibiotics when specifically indicated [5, 6]. In addition, Yemen introduced the Rotavirus vaccine as part of the routine childhood immunization schedule in 2012 [7].

The most recent nationally representative survey of population health status – the Yemen National Health and Demographic Survey (YNHDS) - was last conducted in 2013, before the start of the conflict. At that time, about 31% of children under-five were reported to have had diarrhea during the 2 weeks before the survey [8]. With an under 5 population estimated at 5.16 million, this could translate into 1.60 million cases of diarrheal diseases in this population at any given time [2]. According to the YNHDS, one-third of children with diarrhea were taken to a health facility or provider for treatment, 60% of children with diarrhea were treated with ORS or increased fluids, and 19% received no treatment either from a health provider or at home [8].

The prevailing insecurity, lack of resources for seeking care, and the deterioration of health systems and services has likely made access to health care services even worse than the situation in 2013 as per the YNHDS. However, as this information is out of date due to the rapidly changing situation, the assumptions that were true pre-conflict are now only partially, if at all, applicable. Therefore, the Ministry of Public Health and Population (MoPHP) and all its partners in the health sector including United Nations agencies and non-governmental and civil society organizations do not have the required information for policy and strategy level decision making, preparing well informed service delivery plans for diarrheal diseases, planning at national and sub national levels for supplies (how much ORS and zinc should be ordered for the whole year, how and where should it be pre-positioned and delivered to districts), and for deployment of human and other resources.

A treatment coverage estimate is thus needed but without electronic data archiving or registration it is impractical for the MoPHP and health partners to survey each district due to limited staffing, travel difficulty due to poor road infrastructure and lines of conflict, and safety concerns. While a cadre of Community Health Workers (CHWs) has recently been launched and is currently in scale-up phase, at the time of writing, the data from this program is not yet accessible to add to the data from health facilities on the number of children detected and treated with diarrhea. Planning of where to place these community-based service providers has been informed by mapping areas that lack access to a health facility, but not by disease burden due to this lack of data on population in need. Ideally, CHWs and health facilities would submit reports on time and detailing the number of cases presenting per month, treatments provided, and treatment outcomes. Population representative surveys would additionally be conducted to assess the level of disease burden and care seeking habits among the population. In the absence of these, an estimate based on available data and evidence-based assumptions is needed.

To tackle this problem and develop incidence and coverage estimates for diarrheal disease among children under 5 years, a computational modeling-based solution is proposed. This model combines the health data that is available on number of consultations provided to children presenting with diarrheal disease; data on conflict events and infrastructure status provided through the Logistics Cluster and weather predictions based on historical data from the World Bank to estimate the burden of disease and the underlying lack of access to care. This in turn makes it possible to locate untreated pockets of children under five suffering from diarrheal diseases [9, 10]. By using the synergies of these data types in a modeling framework, we can replicate the accuracy and reliability of active community data collection approaches without adding to the burden of health workers or exposing them to unnecessary danger in such an insecure environment.

## Methods

**Fig. 1:**
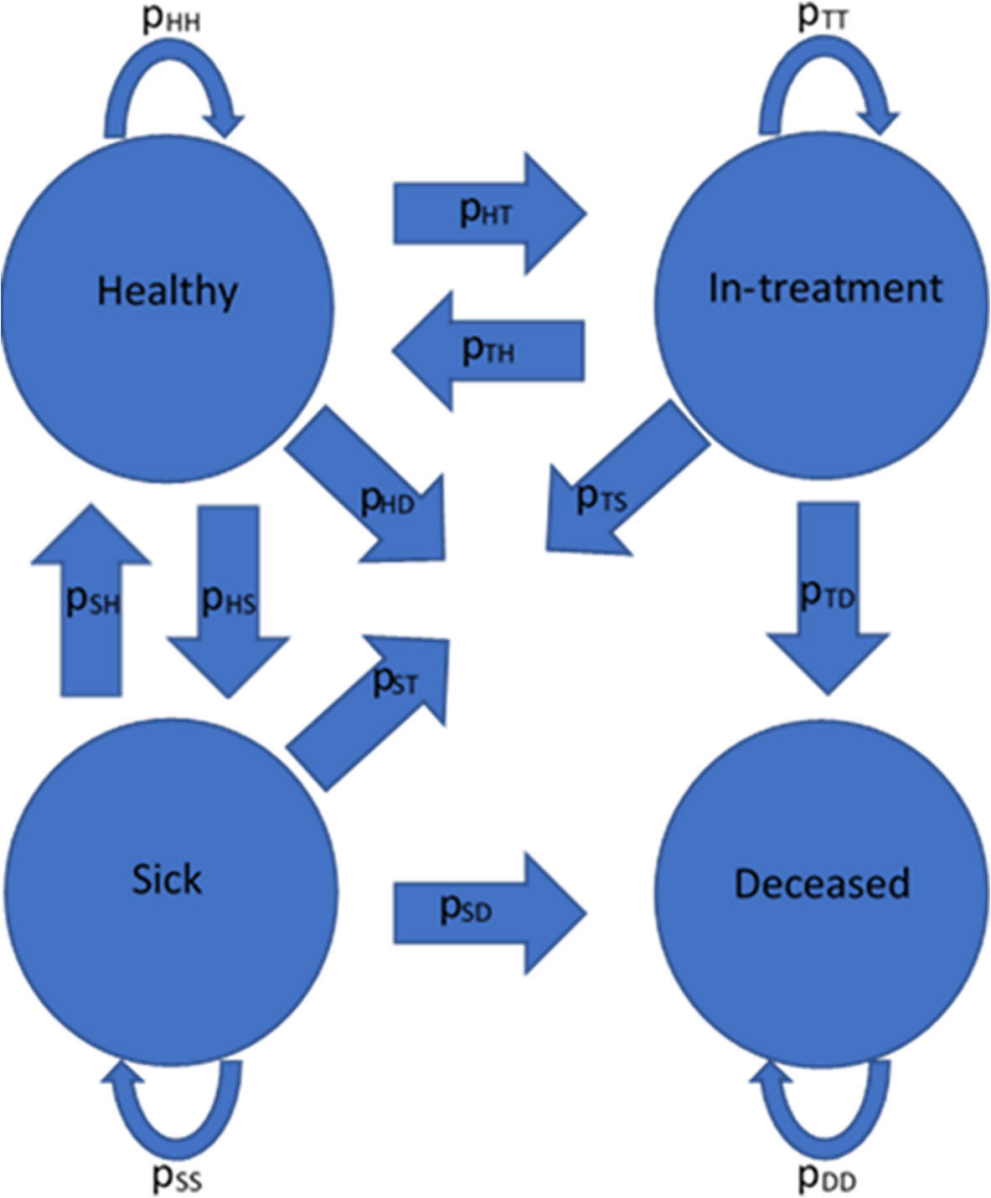
Model schematic. Overview of the possible interactions between the four different health status in the model

As the primary function of the model is to determine the number of children in each health status at a given point in time, the model’s core consists of a basic Markov model. This category of model has been used for various types of probabilistic modeling over the past 20 years, including epidemiological modeling, especially when the model has a defined number of outcomes, or states [11]. The Markov model used for the treatment coverage model for Yemen was composed of four different health statuses (*Healthy, Sick, In-treatment*, and *Deceased)* for children to move between based on a set of probabilistic transition rates as depicted in Fig. 1. This means that by just knowing these probabilities the model can produce a breakdown of the number of children who are estimated to become sick or seek and receive treatment. The model rests on the following assumptions:

1. Any sick person who decides to seek treatment will contact a healthcare provider in community, in a mobile team or at a health facility, and will be treated;
2. To reach the *In-treatment* health status one must have previously resided in the *Sick* health status; and
3. The *Deceased* health status is an absorbing state, meaning once someone enters it, they cannot transition out to another health status again.

These assumptions mean that children will not be turned away for treatment, they must be sick before they can be treated, and if a child dies, they remain deceased for the duration of the simulation. Furthermore, as all children that reach care are assumed to be treated, this means that the coverage rate is really the percent of sick children who are able to reach points of care, as all will be treated.

### Determining the base transition rates

The health facility data collected through UNICEF programs in-country provided insight into the number of cases of diarrheal disease among children under 5 years old that were seen and treated (p_ST_), the mortality rate among children treated with Zn/ORS solutions, the efficacy of Zn/ORS solutions in treating the disease and the percentage of the population the monitored facilities are estimated to have served. As the UNICEF data can only track consultations, our model has been designed to also allow for potential double counting by tracking the number of cases seen and not the number of individual children who fall sick. This information was filtered to examine only diarrheal diseases and to eliminate any incomplete reports where the total number of reported children treated for diarrheal diseases did not equal the sum of all the individual children reported to have diarrheal diseases. Only completed data was used to not skew the value of the resulting transition probabilities and to match the assumption that all who reach a health facility will be treated. Coupled with the most recent overall mortality rates in the literature from the WHO and an estimated incidence rate for Yemen from El Bcheraoui et al., the transition rates were calculated to allow the model’s output to match the previously mentioned program data [2, 12]. The final transition probabilities were as listed in Table 1. An overview of the terms used, as well as their values and meaning are provided in Table 2. These are described in more detail later in the manuscript.

**Table 1:** Transition Rate Probability Table

Health Status Transition	Transition Probability
Healthy to Healthy (p_HH_)	.7601
Healthy to Sick (p_HS_)	.2388
Healthy to In-treatment (p_HT_)	0
Healthy to Deceased (p_HD_)	.0011
Sick to Healthy (p_SH_)	.6968
Sick to Sick (p_SS_)	(1-(Movement function+ 6.2712)/9)
Sick to In-treatment (p_ST_)	(Movement function-.01296834)/9
Sick to Deceased (p_SD_)	.001441
In-treatment to Healthy (p_TH_)	.9902
In-treatment to Sick (p_TS_)	.00961
In-treatment to Treated (p_TT_)	0
In-treatment to Deceased (p_TD_)	.00019
Deceased to Healthy (p_DH_)	0
Deceased to Sick (p_DS_)	0
Deceased to In-treatment (p_DT_)	0
Deceased to Deceased (p_DD_)	1

List of the probabilities of a child transitioning from one state of health to another in the model

**Table 2:** Table of Terms

Object Notation	Summary	Value
Susceptible Population	Total under 5 child population	358,498
Cycles	Number of weeks the simulation runs for	52
Maxroad	Max value of roads	variable
Roadmin	Minimum value of roads due to washout	equation
Bridge	Percent of open road segments with open bridges	variable
Nonbridgemin	Minimum road value with operating bridges	equation
Bridgecuttoff	Weather threshold where lower quality roads may begin to experience washout	0.5
Scalemax	Parameter determining how long infrastructure stays at max value	variable
Weatherweight	Weighting of weather on transition rate	0.3
Roadweight	Weighting of infrastructure on transition rate	0.7
Y(t)	Weather function	Equation
Road(t)	Road and bridge infrastructure function	Equation
YT(t)	Transition probability equation from Sick to Treated	Equation

Overview of the variables and parameters used throughout the model

### Relationship between incidence rate, rotavirus and healthy to sick transition rates

An incidence rate of seven episodes of diarrheal disease among children under five per person-year estimated by El Bcheraoui et al. was used as the initial assumed metric for the healthy to sick transition rates to match [2]. This was then modified to account for general sources of diarrheal diseases and in particular Rotavirus, the most common source which is noted as the cause of between 35 and 60% of enteric diseases in children under 5 years old [13]. Rotavirus infections confer natural immunity, protecting against 87% of subsequent severe diarrhea cases which increases with each subsequent infection [13]. Thus, for n rotavirus infection after the first infection from rotavirus the likelihood that a child is not protected against severe diarrhea can be roughly estimated at a probability of 0.13^n^ [13]. With these factors implemented the general transition rate from healthy to sick with Rotavirus or non-Rotavirus diarrheal disease was adjusted to attempt to match previously estimated incidence rates as discussed further in the validation section.

### Variables and parameters used throughout the model: weather, conflict and their combined effects on transportation infrastructure

The health information system in Yemen was weak even prior to the current crisis, with a reliance on paper-based reporting and challenges with completeness and timeliness of data received at governorate and national levels. With the onset of the conflict and humanitarian crisis which has now brought the health system to the brink of collapse, these gaps in the available health system data are even more notable. This issue affects the data on childhood diarrheal disease as with all other routine administrative data tracked through the health information system. To mitigate this concern about quality of routine administrative data on childhood diarrheal diseases, other types of data outside of traditional health data were incorporated, including the occurrence of conflict events, weather patterns, and the effects of both of these on the traversable nature of transportation infrastructure (roads and bridges). The factors influence p_ST_, the probability determining the likelihood that someone will be willing and able to seek out treatment and thus move from the *Sick* health status to the *In-treatment* health status, and conversely, the probability that a sick person will remain sick via p_ss_ since a larger proportion of sick people seeking treatment should result in a smaller proportion not seeking treatment. The extent of conflict events (represented in the model as the state of the infrastructure) and the estimated weather conditions for that time period allows for the creation of an equation incorporating the time varying nature of these data types into the probabilistic decision process of the model. Thus, the model is not only probabilistic but also time varying.

The state of the weather and the quality of the roads and few bridges in Yemen, which are already affected by and dependent on the state of the conflict and so serve as a proxy for the severity of the conflict, have been shown to be related functions. These factors were selected due to the importance that climate change (in the form of precipitation and storms) and conflict (in the form of destroyed transportation infrastructure and unwillingness or inability to cross lines of conflict) have to the average Yemeni. The weather component is assumed to be a sinusoidal function with a period of 1 year. This weather function was created as a normalized function of best fit from monthly precipitation data for Yemen from 1901 to 2016 published by the World Bank [9]. Clear weather will not alter movement but as rainfall increases travel becomes more difficult and therefore less likely. For our model this means the range of unweighted probabilities could vary between 0, which in the context of the model would indicate a perfect storm during the heaviest rainfall of the year preventing anyone from traveling or reaching their destination, to 1, which would result in anyone being able to access roads leading to health services during the driest week of the year. A constant threat of airstrikes and fighting will further reduce this function. Based on data published by the Yemen Data Project Organization indicating that there are 21.6 airstrikes for every 1000 people, it is further multiplied by a set constant of 97.84% [14]. This probability represents an individual’s likelihood of not being in an airstrike as there will be .0216 airstrikes per person. If viewed as a percent, then on average a person has a 2.16% chance of being affected or a 97.84% chance of not being affected. Like the rains, this can deter people from traveling and further destroy infrastructure, albeit much more directly.

It is estimated that Yemen has about 50,000 km of roads [15]. As the country does not have a functioning rail system to aid in transportation, these roads are the primary means of transportation for people within the country [15, 16]. Despite the importance of these roads, reports from the World Bank estimated that only 28% were all-weather paved before the conflict’s start, with only 11% of rural roads being paved [15]. These nonpaved road segments have been reported by USAID to be damaged both by airstrikes and seasonal rains which has led to flooding, hindering travel during the rainy season [17]. The model was constructed on the assumption that the rains predominantly affect the coastal regions to the south and west of the country and so only 50% of the unpaved roads potentially experience wash out from the worst of the rains. The bridges have also been affected by both the weather and the war: air strikes and fighting have destroyed some bridges, while many others have their access restricted, according to maps published by the World Food Program and the Yemen Logistics Cluster [10]. These restricted bridges were noted to have detours that “may not be accessible during the rainy season” [10]. Thus, when the rains begin the weather function discussed previously will start to decrease. When the weather function drops below a tunable threshold called “bridgecuttoff” in the model, a percentage of the roads corresponding to passable bridge detours that were previously opened will close. This mimics the idea that once the rain intensity increases to this specific threshold, routes that were previously accessible due to dry season detours around destroyed bridges will no longer be traversable, decreasing the number of open routes. The percentage of bridges that would close was designed to scale with the weather and vary from its maximum and minimum values due to the conflict, providing an alternative to “all or nothing” values for the bridges.

Different sections of the same continuous road may have different conditions, with some sections of the same road being in better or worse conditions than others. To improve specificity, the roads shown on the maps published by the Logistic Cluster and World Food Program were subdivided into individual road segments. A road segment was defined as a unique stretch of road that connects between marked communities or another road segment. Each segment could be either open (traversable) or closed (non-traversable). The percentage of open road segments is the output of the Road function. This function is composed of the first five numbered functions below.

Equation 1 determines the maximum value the road function can produce given the conflict and weather. The maximum value will occur when the weather is clear and there are no restrictions due to bridges or the associated detours being closed from rain. However, while its maximum value occurred when there were no weather based restrictions on travel, the conflict is still ongoing. As this is a significant factor in the state of travel infrastructure, the max road value was ultimately determined from the extent of the conflict. This *Roadmax* value serves as a proxy for the severity of the fighting as these values were based on how extensive the conflict was during the period examined; the weather provided more regular variation determining how useable these open road segments were.

1 $$ Roadmax=1-\left(\% closed\ road\  due\  to\ conflict\right)-\left(\% road\ with\ impassable\ bridge\right) $$

With the base road segment conditions from the fighting set in eq. 1, the minimum road segment value was calculated according to eq. 2. This value was the percent of road segments left open from the conflict (*Roadmax*) being directly degraded further by weather conditions, specifically heavy, seasonal rainfall. This was calculated as the percent of road segments left functional after the assumed max wash-out of the previously open road segments, minus the percent of road segments that have a dry season bridge detour as the heaviest rains would make these detours unusable.

2 $$ Roadmin=.64\times Roadmax- bridge $$

As previously noted, the state of all the bridges are not simply purely opened or purely closed. Based on the severity of weather, the percentage of these bridges that are inaccessible will change. As weather worsens beyond a point, more bridges and detours will close and vice versa. This is only true for severe weather events. Bridges will retain their normal degree of functionality up to a certain degree of weather severity, the tunable “bridgecutoff” value discussed previously. This leads the function depicting the number of bridges down to have different behaviors depending on weather conditions as shown in Eq. 3.

3 $$ \mathrm{Bridgedown}(t)=\left\{\begin{array}{c}\left(1-\frac{1}{bridgecutoff}\right)\times bridge\times \mathrm{Y}(t),\mathrm{Y}(t)\le bridge cutoff\\ {}0,\kern14.1em \mathrm{Y}(t)> bridgecutoff\end{array}\right. $$

With these components all determined, the road function could be fully built and is described in Eq. 4. The minimum function (min) in Eq. 4 ensures the Road function never produces a value beyond its maximum allowed value as determined by Eq. 1. All previous equations are contained within this function including the best fit weather function Y(t). When combined with a scaling factor this function allows for user input, allowing it to be more finely tuned beyond the national average if needed.

4 $$ \mathrm{Road}(t)=\min \left\{\left(\left[\mathrm{Y}(t)\times \left( scalemax-\left( Roadmin+ bridge\right)\right)+\left( Roadmin+ bridge\right)\right]-\mathrm{bridgedown}(t)\right), MaxRoad\right\} $$

While the roads are a major factor in a person’s willingness to travel to seek treatment, the weather too may play a role in this decision. As such, the overall transition probability movement function YT(t) was then created as a weighted average of the weather function, Y(t), and the infrastructure and conflict function, Road(t), as seen in Eq. 5. As YT(t) is a component of a probability, the weighting was necessary to ensure the sum of the weather and road functions never resulted in a case of greater than 100% probability.

5 $$ \mathrm{Y}\mathrm{T}(t)= weatherweight\times \mathrm{Y}(t)+ roadweight\times \mathrm{Road}(t) $$

### Combining transition rates and variable parameters to estimate treatment coverage for children with diarrhea

With the weather and conflict (i.e. road) components combined to form the transition rate function for the probability that a person will travel to seek treatment and thus change from the *Sick* health status to the *In-treatment* health status, it became possible to estimate the coverage of treatment services for children under five with diarrheal diseases. From the assumptions, each sick person that reached a treatment center (p_ST_) was treated with Zn /ORS. As this transition occurred only after a person became sick, the estimation of those seeking treatment (the coverage rate) was calculated from Eq. 6.

6 $$ \mathrm{Coverage}(t)=\frac{In- treatment\left(t+1\right)}{Sick(t)} $$

The model is designed that it takes one iteration (i.e. one simulated week) for a sick child to move into the *In-treatment* health status. A subset of the *Sick* population at week t would move to *In-treatment* at week (t + 1). Thus, to calculate the treatment coverage of sick children, the *In-treatment* population at week (t + 1) must be divided by the *Sick* population from week t.

### Determining the model’s population size

To ensure the model output accurately matched the UNICEF provided data, the model’s population size was next determined. The under-five population was estimated at 5.16 million children [18]. Half of this population was unable to reach a fixed health facility before the war started [2]. With this in mind, these pre-conflict health facilities were assumed to be all functional as there was no conflict to close them. To determine what proportion of this 50% of the population was reaching care during the conflict we next examined the health facilities that were open during the conflict. According to the HeRAMS data, 45% of health facilities are fully functional and 38% are partially functional. For the purposes of this model, it was assumed that a partially operating health facility was roughly equivalent to half of a fully functioning one, and thus 38% partially functioning facilities is equivalent to 19% fully functioning. This leads to a final estimation of 64% functionality of health facilities. 53% of the facilities in the data set on facilities treating children provided by UNICEF had complete data on the number of diarrheal disease cases they saw for children under 5 years old. This data set consisted of about 41% of all the primary care facilities monitored by UNICEF. This information was combined to estimate the population size that has been able to reach health facilities on the ground and produce the primary data. In order to finalize transition rates in the model and still match the data outputs, the model would also need to use this same sample size which was estimated at 5,156,951 × 50% × 64.26% × 53% × 40.82% = 358,498 children.

### Deterministic sensitivity analysis

A 1-way simple, deterministic sensitivity analysis, one of the most widely used methods to study model uncertainty, was then performed for “maxroad”, “Roadmin” “Bridge”, and two parameters from the function Y(t), namely the conflict multiplier and the weather amplitude [19, 20]. This analysis is useful as by examining the stability in the model and its parameters, we can further improve confidence in the model [21]. For this 1-way deterministic analysis, each parameter was modulated between 10% and 200% of its base value, shown in Table 3 except for the weather amplitude which could only be increased to 190% due to probability constraints in the model. The model estimation of the number of children seeking care was repeated the same way as described above for each parameter throughout the sweep, identifying which pieces of data had the greatest impact on both the maximum weekly coverage and minimum weekly coverage.

**Table 3:** Sensitivity Analysis Parameters

Parameter	Base Value	Values Swept
Maxroad	.4430	.0443–.886
Roadmin	.2479	.0248–.4958
Bridge	.0804	.00804–.1608
Air Strike Proportion	.0216	.00216–.0432
Weather Amplitude	7.174	.7174–13.6306

Summary of parameter baselines and ranges swept for a 1-way deterministic sensitivity analysis

## Results

### Validating the model

**Fig. 2:**
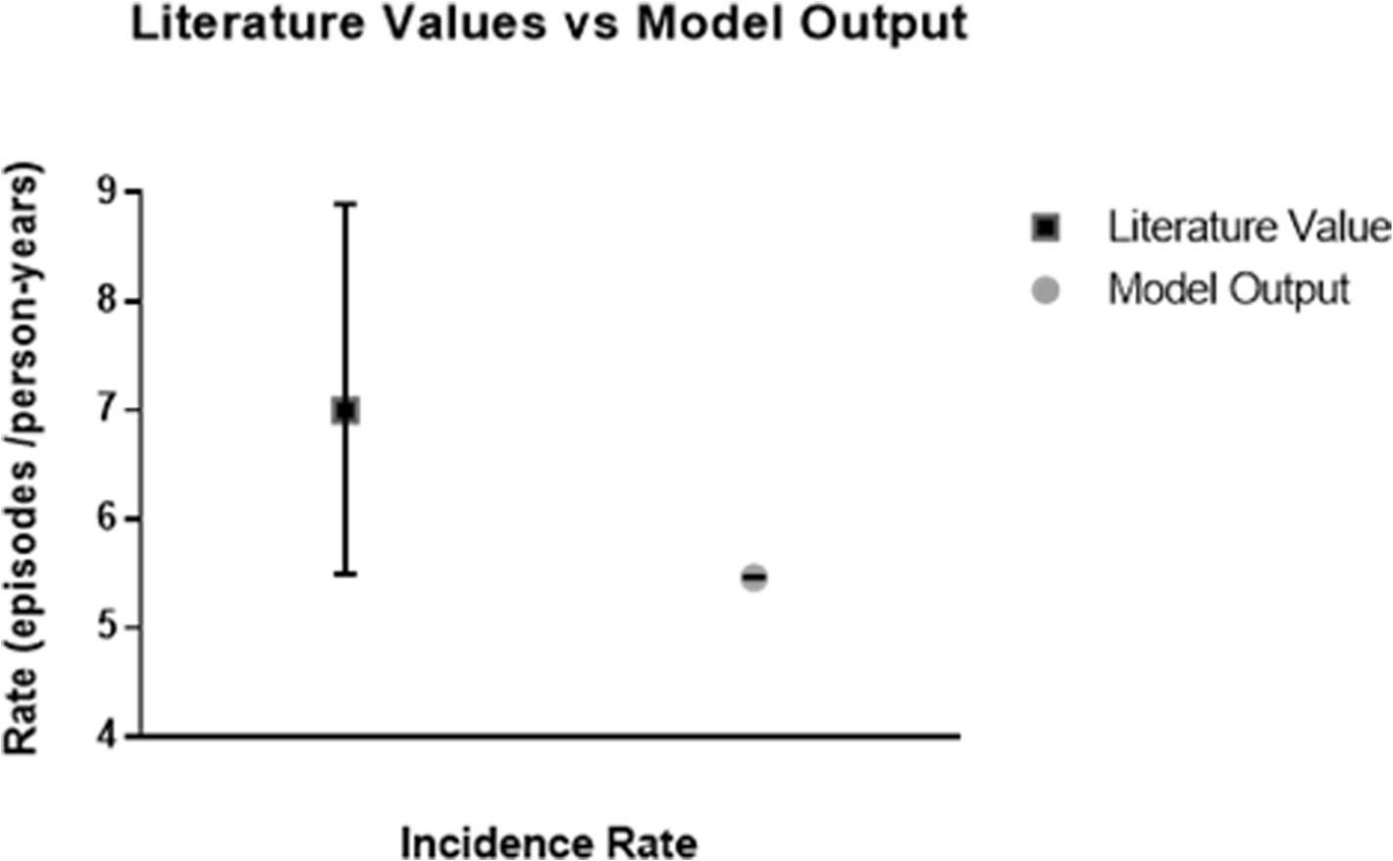
Incidence rate comparison. The comparison between data published by El Bcheraoui et al. and the average output of the model after 7 iterations for 2016. Error bars represent the upper and lower bounds of the 95% confidence interval analyzed in GraphPad. Although numerically close, the model’s output for the incidence rate is deemed to be statically different as it falls outside the confidence interval of the target estimation data

The results of the validation data tests were first examined. Running the model for 1 year from January through December produces overall values for the under-five general mortality rate that is slightly higher than WHO estimates for all-cause mortality and an incidence rate below a prior estimated value from the literature. These results affect all death rates (p_HD_ + p_SD_ + p_TD_) and p_HS_ respectively. Table 4 compares the average all-cause under-five mortality rate (p_HD_ + p_SD_ + p_TD_) of the model run seven times against the published probability of mortality for children under five in Yemen for 2016. For a sample population of 100,000 children under five, the Markov model for Yemen estimates an excess 340 deaths compared to the WHO estimate averaging over the male and female deaths. This average was selected as the UNICEF provided data and the model results are aggregated and not specified by gender like the WHO data. Figure 2 depicts the comparison for the incidence rate of diarrheal diseases for this same time period. This figure shows that despite the large 95% confidence interval (7 incidneces/person-year, CI: 5.5–8.9 incidences/person-year) of the estimated incidence rate for 2016, the most recent year estimated, the model’s incidence rate is lower (5.465 incidences/person-year). Although this was the latest year estimated, given the dynamic nature of the crisis, it is not surpising to see a different incidence rate leading to the validation of the programatic data than what was published 2 years prior to the primary data.

**Table 4:** Model validation against published 2016 WHO Life Tables for Yemen

2016					
WHO Global Health Observatory Data Repository				Mathematical Model	
Indicator	Age Group	Male	Female	Age Group	All Children
Cumulative probability of dying	< 1–4 years	0.05745	0.05152	< 5 Years	.05769
Cumulative number of people dying	< 1–4 years	5710.947	5146.47	< 5 Years	5769

The WHO section on the table’s left shows aggregated values for children under 5 years old, providing an overall mortality probability as well as the number of children dying throughout the year based on a hypothetical birthrate of 100,000. The Mathematical Model section in the two rightmost columns examines the aggregated values from the model using the same hypothetical population size

Further discrepancies between the model and the UNICEF provided data can in part be attributed to uncertainties in the data due to the situation on the ground and the difficulties in collecting accurate data in the country while the WHO estimations may vary due to being based on other models and not resulting directly from primary data. To this end, the data used has been selected as the most complete and verifiable. That said, to help account for how different conditions may impact the model’s findings a parameter sweep was also performed and its results will be discussed later in the text.

**Fig. 3:**
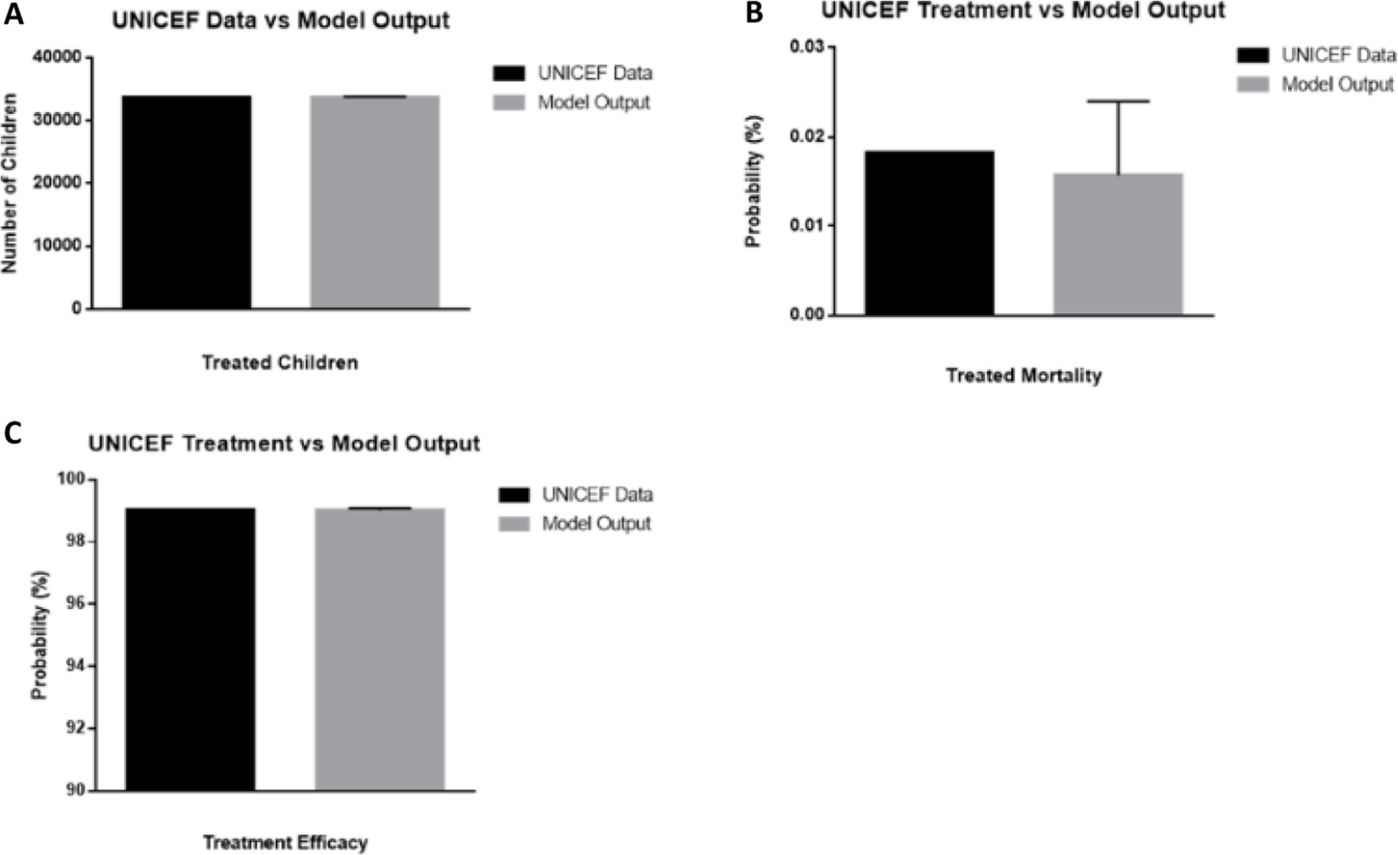
Comparison to Number of Children Treated (**a**), Treatment Mortality (**b**) and Treatment Efficacy (**c**). Comparison of the model against validation data showing no significant difference between the **a** total number of children under 5 treated (*P* = .8864) and **b** the treatment mortality rates from 2018 health facility monitoring data collected through a third party (*P* = .4479). **c** The efficacy of Zn/ORS intervention from the same data set (*P* = .8450) was also not deemed to be significantly different than the model. Significance was assessed with a one sample T test in GraphPad. Error bars shown as standard deviation for *n* = 7 code iterations

With the inclusion of the new infrastructure, conflict and weather information, the model could recapitulate the observed mortality rate of children that were treated for diarrheal diseases averaged across the 463 facilities reporting complete data for the five-month period ranging from May to September 2018. A side by side comparison of the model and the metrics used for validation is provided in Fig. 3. From this comparison, it was observed that the model’s tracking of the number of children reaching care and being treated was also in agreement with the health facility data collected through UNICEF’s program data. This provided sufficient evidence for successful validation of p_ST_, p_TH_, and p_TD_. After comparing the model’s output against the WHO estimates and UNICEF’s programmatic data, and finding a sufficient match, the model was viewed as sufficiently validated and the base transition rates used to create this validation were set.

### Conclusions on the validity of the model

**Fig. 4:**
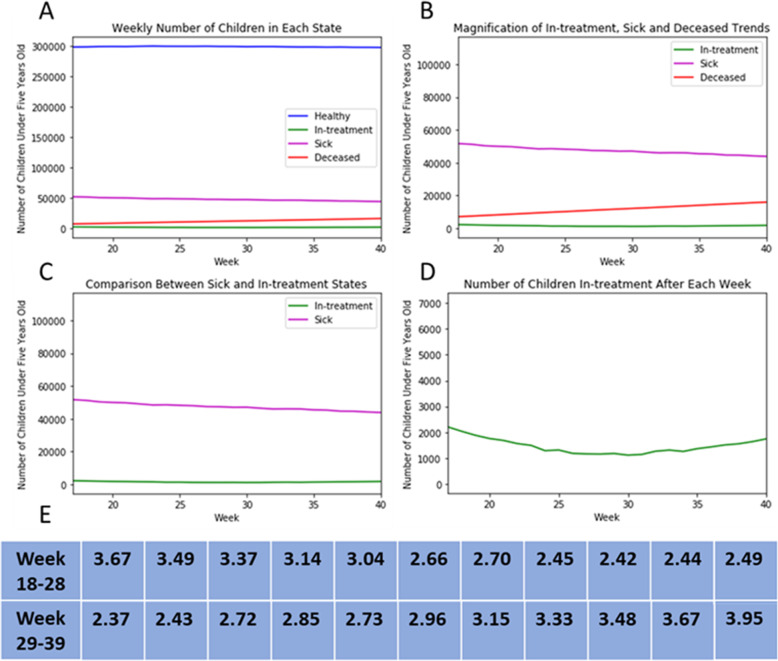
Health trends for children under five in Yemen during May–September of 2018. **a-e**. The week number is the number of weeks since the start of the year. **a** Overall weekly distribution of the health-states for the estimated child population and **b** the zoomed in to see the bottom half of the graph. **c** Plot of the total *Sick* health status population vs time compared against the *In-treatment* health status vs time, with the *In-Treatment* health status enlarged in (**d**). **e** The coverage rate was listed week by week

With validation complete, predictive estimates for the treatment coverage rate, or more specifically the percent of sick children reaching, and thus receiving, treatment were then able to be made with an increased degree of confidence. The rate of those seeking care was estimated on a weekly basis by comparing the newly validated number of treated children at each time-point and dividing by the total number of sick children according to the model. The first weekly coverage assessment was during the same time as the 2018 validation data. These predictions yielded the following treatment coverage rates and trends shown in Fig. 4 for a population matching the disease prevalence of 31% from the last National survey conducted in 2013.

### Sensitivity analysis

**Fig. 5:**
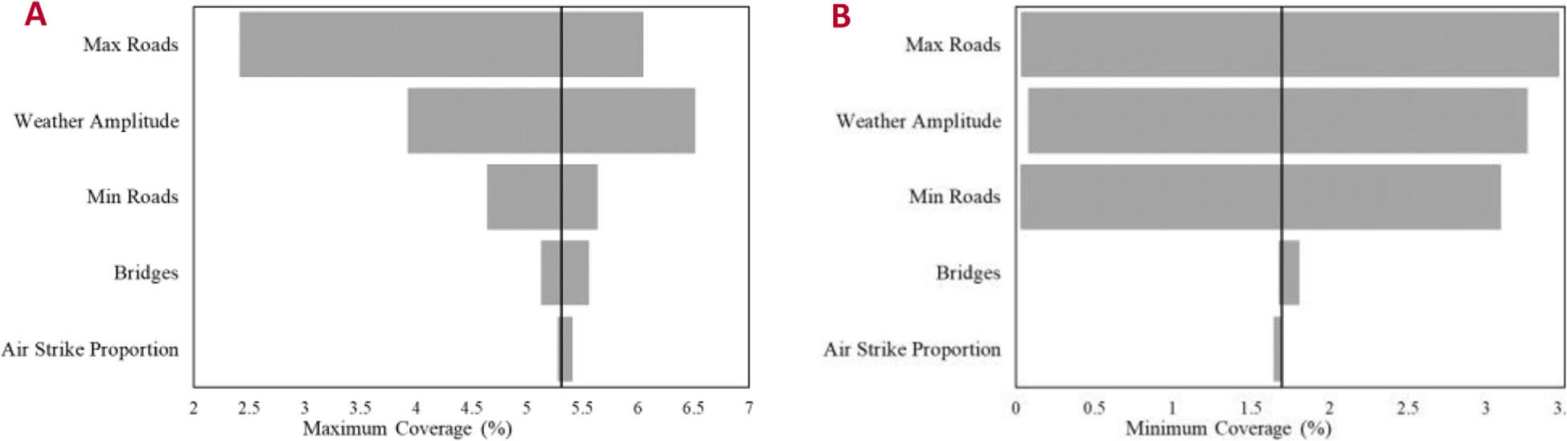
Deterministic Sensitivity Analysis for the maximum estimated coverage (**a**) and the minimum estimated coverage (**b**). Parameters were swept from 10 to 200% of their baseline values except for the Weather Amplitude, which was varied from 10 to 190% due to larger values producing unrealistic probabilities (greater than 1 or less than 0). The three most sensitive parameters were the maximum road value, weather amplitude and the minimum road value in all cases. **a** The maximum coverage was more sensitive to decreasing values compared to the minimum coverage (**b**) which was equally sensitive to parameter changes in both directions

As the model was dependent on data from the ground that can be hard to accurately measure due to the conflict, the values used for the parameters influencing the number of sick children who would seek and ultimately reach care, were varied in the deterministic sensitivity analysis, helping to improve the generalizability of our findings [20]. Fig. 5 showed the parameters that have the most influence on the number of children reaching primary care facilities (and thus the estimated coverage). Larger bars represented a bigger impact on coverage while the vertical black line represented the baseline coverage for both the maximum estimated coverage (5.31%) and the minimum estimated coverage (1.70%). Small bars represented parameters that do not have as much impact on the coverage compared to larger bars. In both cases, the parameters that had the largest impact were the maximum value of the road, representing the percent of road segments currently traversable despite the effects of the conflict, the severity of the weather noted as the weather amplitude and the minimum value of the roads due to closures from both conflict and weather.

### Looking forward: capability of the model to be used for policy-setting and health service planning

**Fig. 6:**
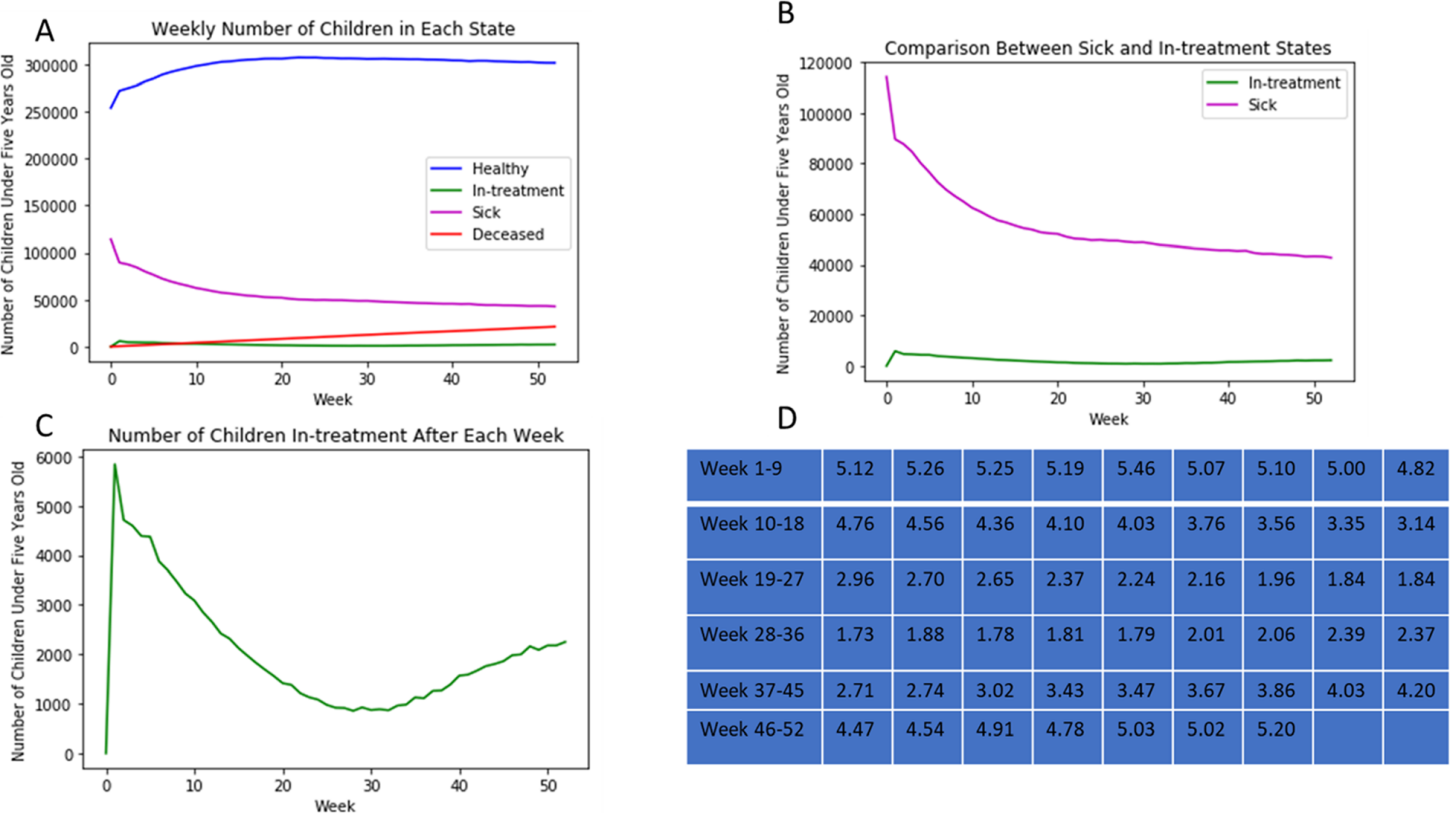
Predictions for each state (**a**), no *Deceased* and no *Healthy* (**b**), or *Sick* states (**c**) and weekly coverage rate (**d**). **a** Tracked changes over all health status. **b** Comparison between *Sick* and *In-treatment* health status throughout 2019 and **c** a magnified view of the *Sick* health status. **d** The weekly percent treatment coverage during the year

While useful to retrospectively assess treatment efficacy, the model is more powerful when used to predict future trends and treatment coverage rates so that practical policy decisions can be implemented. By knowing how successful an intervention will be in advance and when there will be an increase in children seeking and needing care, the national and local health authorities, with the support of aid organizations, can select the policies that maximize the number of children treated, leading to an increase in coverage of children reaching care. Considering this, Fig. 6 shows the predictions made for 2019. These novel, post-validation results show that the weeks with the lowest coverage rates coincided with the middle of the year during the rainy season. For 2019, it is expected that the average country-wide weekly coverage will be below 2% for about 8 weeks, with a peak in coverage of about 5.1–5.2% of the population of sick children under 5 years old with diarrheal diseases seeking and receiving treatment near the beginning and end of the year. However, as previously stated, since the model assumes all who reach a health facility are treated, this low number indicates that this coverage estimation is the percentage of sick children who are seeking out and accessing treatment. As shown in Fig. 6, this decrease results from the fact that despite the decrease in the overall sick population, the decrease in the number reaching treatment occurs more quickly, leading to the observed change in coverage. Thus, future efforts would have the largest room for improvement during this time as the number of sick children that need treatment remains high and the least number of these children are being treated.

## Discussion

The extent of the conflict’s effect can be seen in a comparison between the same week across different years. As the weather is assumed to follow the same general pattern between years, the only difference between the same week during two different years is the state of the conflict. As fighting continues and the infrastructure is further damaged or otherwise inaccessible, the values of the road maximum, minimum, and bridge values are decreased. This results in a decrease in probability that a sick child with a diarrheal disease reaches a primary care facility and thus ultimately transitions from the *Sick* health status to the *In-treatment* health status. This is seen in the week 28 value for 2018 of 2.49% in Fig. 4 decreasing by about 31% over the following year to 1.73% as shown in Fig. 6. In these cases, the extent of healthcare coverage, specifically the number treated, and the value of the transition probability movement function, YT(t), serve as indirect measurements of the conflict’s worsening effect on the health of children in Yemen.

The most noticeable trends are the seasonal nature of those seeking treatment and the decaying exponential behavior of the overall number sick. The weekly number of children treated at a primary care facility closely follows the sinusoidal nature of the roads and weather. This makes sense as these were the main factors influencing this transition probability and what the model is most sensitive too, though it is not exact as the overall population of children in the model continues to decrease due to a combination of the absorbing nature of the *Deceased* health status and the shrinking *Sick* population that directly feeds this health status. While the overall *Sick* population decreases in a manner similar to an exponential decay curve, it begins to abruptly slow the rate of decrease around the 20th–25th week, around the same time that the *In-treatment* population begins to increase. The transition point where the slope of the *Sick* curve is lowest indicates that the number of weekly diarrheal cases has increased to nearly match the number of children leaving the *Sick* health status; the number of children becoming sick nearly matches the number recovering or seeking treatment. This inflection point occurs during the time that the average rainfall historically is at or near its peak. Despite this increase in the number of new diarrheal cases, the number seeking treatment does not increase proportionally. As healthcare coverage is defined as the number of children residing in the *In-treatment* health status in a week divided by the number of children in the *Sick* health status from the prior week, this unequal change leads to a period of decreased coverage. Beyond this point, the model depicts movement in the opposite directions for the number being treated and the number sick. This anti-parallel movement appears to make intuitive sense; if more people are treated there should be fewer sick children as they will leave the *Sick* health status to transition into the *In-treatment* health status. This trend can also be explained by the fact that the pathogens that cause diarrhea are often water-borne and thus disease is seasonal with rains, while the ability to access treatment would decline as road networks deteriorate at the same time. Conversely, during dry seasons, fewer diarrheal disease cases would be expected because transmission factors are reduced, and those who are sick would be better able to access treatment as road networks are more traversable. Although there is a greater numerical change in the number of children residing in the *Sick* health status compared to the *In-treatment* health status, the percent change in the *In-treatment* health status seems to be more drastic. This could indicate that although more children may be helped by slowing the infection rate, improving access to care via minimizing the effects of the weather and travel restrictions will lead to a greater percent change for the number of children receiving treatment.

It is also worth noting that despite the years of war, the overall disease burden represented by the number of children in the *Sick* health status seems to have decreased. Although the YNHDS value of 31% was used in both simulations as the initial prevalence of the disease, the number of sick children rapidly dropped at the start both times, seeming to indicate that the actual value may be closer to about half as much, a testament to the work done by all health actors in the region that have improved conditions such as the tripling in number of people reached by water, sanitation and hygiene (WASH) efforts between the months of May and August [22]. As the model predicts, this will be the period when there is an increase in the amount of contaminated surface water due to the rain so these preventative efforts are especially important during this time and this policy trend should continue. This serves to decrease the burden of disease and the ultimate population in need as shown in the model. While critical for this work to continue, especially in order to achieve a continued reduction in the prevalence of these diseases, these burden-decreasing approaches do not directly address those that are already sick and currently in need of treatment which is an important aspect of the coverage rate. As the population currently being treated is smaller than the sick population in need of treatment, an increase in the number receiving treatment will have a larger effect on the coverage compared to an equal size change of those in need. As Fig. 4c shows, at the lowest point an additional 1000 children reaching care and being treated throughout Yemen in 1 week would nearly double the coverage rate, but in Fig. 4b, 1000 fewer children becoming sick at this same time would only result in about a 2% increase in percent healthcare coverage due to 1000 children being a much smaller proportion of the nearly 50,000 sick children than the 1000 treated children.

Based on this set of observations, it seems that the best way to increase the number reaching care would be to enact policies that increase treatment efforts and access during the peak periods of rain around the middle of the year. This might best be accomplished by focusing on community outreach efforts during, and just prior to, this time since the disease burden is expected to increase while at the same time a smaller proportion of children are reaching facilities for treatment compared to other parts of the year. This could allow for an increase in medication to be delivered in a pre-emptive fashion to outreach programs, helping to keep the number and severity of cases low as people would not have to endure the poor roads and weather to reach medication-based treatment. While the conflict events are more difficult to predict than the weather patterns, similar approaches focused on bringing points of care as close to communities as possible and training and rolling out community-based primary care providers throughout the country will also serve to improve communities’ resilience and mitigate the negative impacts of conflict events on access to essential health services. Having a defined period where outreach is increased serves to be more cost effective compared to increasing these efforts during the same period in the dry season when they may have less of an impact on the coverage or year-round where the operating cost would be increased. Ultimately, a dual approach that leads to an increase in outreach and community-based workers especially during the periods of decreased travel abilities and an increased focus on service delivery through fixed facilities when travel conditions improve, should be utilized. Given the full year prediction for 2019 produces similar trends, this also seems to support the fact that an additional treatment approach should be utilized to improve the treatment coverage of Zn/ORS for diarrheal diseases in children under five. Without this change, potentially up to 98% of sick children will not be able to reach and receive the treatment that they so desperately require.

When the values found in both the data provided and the literature were compared against the model’s output, there was no statistically significant difference found between the model and the primary data provided by UNICEF. Among all the data, only the difference between the model’s incidence rate and the estimated literature value was deemed significant. Although care was taken to examine information from different sources to limit errors, the primary data metrics comes from one source and are not without limitations, which could be a source of discrepancy. Along with the previously mentioned fact that 47% of the clinical registry data was deemed incomplete and not used due to mismatching total values, the number of primary data sources was limited due to the conflict itself. Furthermore, each metric was limited in the information it could provide. For example, the incidence rate and overall mortality rate published are for 2016 and are outdated, potentially explaining the discrepancy in our model. These changes in the incidence rate between 2016 and 2019 are likely to have been influenced by a variety of factors. Continuation and increased intensity of the conflict over the years may have led to an increase in both values, although increased efforts by health sector partners including UNICEF to expand functionality and accessibility of health services could have improved treatment rates, and programs aimed at rehabilitating water and sanitation networks could have contributed to decreasing incidence. Due to the scarcity of reporting from the region due to the conflict and the weak information systems, more recent published data has not been found.

Monitoring the effects of Zn/ORS on the course of the disease is also difficult to assess, calling into question the exact efficacy of the treatment. Although it is well established that this is the best course of treatment for those who suffer from diarrheal diseases, its exact efficacy in the region is unknown. Given that this treatment for the majority of diarrheal cases does not require patients to remain in a health facility under the care and observation of a health worker, it is unknown the extent to which children adhere to the global childhood diarrhea treatment guideline of 10 to 14 days of 20 mg doses of zinc for children between the ages of 6 and 59 months or 10 mg doses for the same duration of time for children under 6 months old [23]. It is also not recorded if these children received some other type of treatment from an alternative source during this time. Both factors could affect treatment recovery and mortality rates in the data.

Although this type of model has been used for epidemiology predictions in the past and the model presented does produce similar values to those seen in the literature, it too is not without limitations. By examining Yemen as a whole, population movements are neglected from the analysis. Migrations within the country could lead to regional effects as a sick child becoming infected and receiving treatment in different regions would lead to a decrease in the coverage in the area where the child was infected and an increase in the coverage where the patient was treated. Internally displaced person (IDP) status is also associated with poor living conditions including poorer hygiene and sanitation and limited access to health services, potentially increasing both the occurrence of under-five diarrheal disease and the risk of mortality from this condition in areas where large populations of IDPs are living.

Given that the model was designed to be easily tunable and applicable to other areas of conflict with suboptimal data quality and quantity, future iterations could look at expanding the model into these regions or other diseases / conditions in Yemen. In the current version of the model, the only information required is time series data on a region’s transportation infrastructure, reports on the number of people in a subpopulation treated for the disease (or types of disease) and the outcome of their treatment. With this information, the model can be combined with publicly available information (such as population size and mortality) and provide predictions for a disease or age group in question. Expanding the model into other regions, especially on a subnational level or other diseases could even serve as a further means of validation, thus improving its functionality not only in each new region, but in all areas where it is being used.

As the model is applied to different regions, it is important to examine model limitations through how sensitive the model is to the available data in each region. While the model was validated for a national level overview of Yemen, we note that conflict data, especially its influence on transportation can result in values that alter the maximum output by over 45% and the minimum result by about 100%. Thus, the model is especially sensitive to this data and variations in this input data will result in variations in coverage estimations.

Examining the assumptions that the model rests on also can reveal additional limitations. The assumptions that everyone who makes it to a facility receiving treatment while medical supplies last is one aspect that may cause the model to slightly diverge from the realities on the ground. Due to the ever-changing conditions on the ground, acquiring data in a timely manner that meets the necessary criteria to be integrated into the model is extremely difficult. While this difficulty is one of the initial motivators of the model’s creation, it also serves as another key limitation in the model’s predictive power. By only looking at the data where follow ups occurred to paint the most complete picture, as well as the other assumptions made throughout, it is unsurprising that the model had discrepancies. These are most noticeable in the comparison between the oldest data set, 2013 YNHDS document which stated that in the 2 weeks prior to the survey it was discovered that 33% of the sick population went for advice or treatment from a health facility or provider and of that group 60% were treated with Zn ORS for a total of about 20% of this sick population receiving appropriate treatment over a 2 week interval [8]. The model however underestimates this number and shows that between 3.61 to 10.65% of the sick population received the appropriate treatment of Zn/ORS over a two-week period. Despite this discrepancy, and the fact that this survey depicted conditions before the conflict began, the model still can provide useful and usable information.

Despite these limitations, this model still can be a unique and helpful tool to healthcare workers in humanitarian and conflict settings like Yemen as this model goes beyond past modeling approaches. In the past, infectious disease modeling has largely focused on the deterministic spread of an infection without regard for spatial parameters [24]. These models, especially traditional Susceptible, Exposed, Infectious and Recovered (SEIR) models, are largely deterministic, governed by strict, static differential equations that overlook the inherent randomness of the world, especially the war-ravaged world of a child and often assume homogeneity in the region examined, a factor that is often not true on a large scale [24, 25]. This has, in part, led to more recent agent-based models that include geospatial factors, which are critical to providing more predictive and realistic outcomes and the inclusion of spatial structure into SEIR models [25, 26]. This however often requires “orders of magnitude more data to fit the model”, a luxury that the data quantity from Yemen does not provide [25]. Furthermore, the inclusion of local context such as war-damaged infrastructure, ongoing fighting, climate and the reality that children will be more susceptible must also be considered. Given the simplicity and inclusion of inherent randomness, a Markov model was selected as the basis of our model which was then further modified with the inclusion of these factors. This allows for better evaluation of healthcare coverage and ultimately provides more and more reliable information on which policy decisions can be based.

To the extent of the authors’ knowledge, at the time of writing, this model is the first to propose a computational model for the state of Yemen’s healthcare for diarrheal diseases in children under 5 years old in concert with the examination of the country’s weather patterns and the impacts of the conflict on critical transportation infrastructure. The novelty of this model is rooted in these non-traditional healthcare factors that have been incorporated into our examination. In Yemen, these climate factors are especially important. As climate change increases, places like Yemen are expected to face particular hardships as these climate variations will result in increased morbidity and mortality due to diarrhea as well as “increased frequency and severity of storms and flooding of the low lying coastal areas … and shortage of the fresh water” which are all factors that our model has shown can further decrease diarrheal disease treatment [27]. Thus the inclusion of these weather factors is of paramount importance now and will be even more so in the future.

## Conclusion

To assist healthcare workers and policy makers alike, a computational Markov model was created for Yemen that monitored the spread of diarrheal diseases in children under 5 years of age. The model was able to recreate various trends recorded in the clinical register health facility data collected by a third party on the number of children treated for diarrheal diseases, as well as for the treatment mortality and efficacy rates, and for data previously published in the literature on the incidence rate and the overall mortality rate of this population. With this viewed as sufficient validation for the model, a predictive treatment coverage estimate was generated for the region analyzed on a weekly basis over the course of a year, showing how many sick children sought and reached care.

Based on this predictive chart for 2019, it was determined that the percent of sick children reaching and receiving treatment varied between 1.73% during the worst of the mid-year rains to 5.2% during the last week of the year. As the number treated is always lower than the number needing treatment, increasing the number in this health-status directly will lead to the most drastic increase in coverage percentage, while also serving to indirectly decrease the total sick population as well. Thus, to attempt to increase this value and prevent a decrease in future years, our model recommends policy makers increase the number of community outreach programs just before and during these weeks of increasing disease burden and declining proportion of the population in need that seeks treatment.

## Data Availability

The data that support the findings of this study are available from UNICEF but restrictions apply to the availability of these data, which were used under license for the current study, and so are not publicly available. Data are however available from the authors upon reasonable request and with permission of UNICEF. Code used to reach these conclusions is available upon request.
